# Pancreatic Endoplasmic Reticulum Kinase Activation Promotes Medulloblastoma Cell Migration and Invasion through Induction of Vascular Endothelial Growth Factor A

**DOI:** 10.1371/journal.pone.0120252

**Published:** 2015-03-20

**Authors:** Stephanie Jamison, Yifeng Lin, Wensheng Lin

**Affiliations:** 1 Department of Neuroscience, University of Minnesota, Minneapolis, United States of America; 2 Institute for Translational Neuroscience, University of Minnesota, Minneapolis, United States of America; 3 Masonic Cancer Center, University of Minnesota, Minneapolis, United States of America; University Health Network, CANADA

## Abstract

Evidence is accumulating that activation of the pancreatic endoplasmic reticulum kinase (PERK) in response to endoplasmic reticulum (ER) stress adapts tumor cells to the tumor microenvironment and enhances tumor angiogenesis by inducing vascular endothelial growth factor A (VEGF-A). Recent studies suggest that VEGF-A can act directly on certain tumor cell types in an autocrine manner, via binding to VEGF receptor 2 (VEGFR2), to promote tumor cell migration and invasion. Although several reports show that PERK activation increases VEGF-A expression in medulloblastoma, the most common solid malignancy of childhood, the role that either PERK or VEGF-A plays in medulloblastoma remains elusive. In this study, we mimicked the moderate enhancement of PERK activity observed in tumor patients using a genetic approach and a pharmacologic approach, and found that moderate activation of PERK signaling facilitated medulloblastoma cell migration and invasion and increased the production of VEGF-A. Moreover, using the VEGFR2 inhibitor SU5416 and the VEGF-A neutralizing antibody to block VEGF-A/VEGFR2 signaling, our results suggested that tumor cell-derived VEGF-A promoted medulloblastoma cell migration and invasion through VEGFR2 signaling, and that both VEGF-A and VEGFR2 were required for the promoting effects of PERK activation on medulloblastoma cell migration and invasion. Thus, these findings suggest that moderate PERK activation promotes medulloblastoma cell migration and invasion through enhancement of VEGF-A/VEGFR2 signaling.

## Introduction

The unfolded protein response (UPR), activated by endoplasmic reticulum (ER) stress, coordinates an adaptive program to preserve cell function and survival under stressful conditions [1, 2]. The UPR is mediated by three ER-resident transmembrane proteins, pancreatic ER kinase (PERK), inositol requiring enzyme 1 (IRE1), and activating transcription factor 6 (ATF6). PERK activation inhibits global protein biosynthesis, but stimulates the expression of certain stress-induced cytoprotective genes by phosphorylating translation initiation factor 2α (eIF2α) [3]. Phosphorylation of eIF2α enhances the expression of growth arrest and DNA damage 34 (GADD34), a regulatory subunit of a phosphatase complex that dephosphorylates eIF2α, by promoting the translation of the cytosolic transcription factor ATF4, which forms a negative feedback to down-regulate PERK signaling [4]. It has been well documented that the UPR is activated in solid tumors due to hypoxia and nutritional deficiency, a common feature of the solid tumor microenvironment [5–7]. Nevertheless, the role of the PERK branch of the UPR in tumor development is controversial [8, 9]. Some studies show that PERK activation facilitates tumor development by promoting tumor cell survival and enhancing angiogenesis [10–12]. Other studies show that PERK activation inhibits tumor cell proliferation and leads to cell apoptosis [13–15].

Medulloblastoma is the most common solid malignancy of childhood [16, 17]. Our previous study showed that the UPR is activated in tumor cells in a mouse model of medulloblastoma and that GADD34 inactivation enhances PERK signaling and facilitates the medulloblastoma formation by promoting angiogenesis through induction of vascular endothelial growth factor A (VEGF-A) [18]. It is known that tumor cell-derived VEGF-A acts on endothelial cells to promote angiogenesis and tumor progression [19]. Recent studies also suggest that VEGF-A can act directly on some types of tumor cells in an autocrine manner, via binding to VEGF receptor 2 (VEGFR2), to promote tumor cell growth, migration, and invasion [20, 21]. Intriguingly, a previous report suggests a possible autocrine role of VEGF-A in human medulloblastoma growth [22]. Moreover, several studies show that PERK activation in human medulloblastoma cells enhances the expression of VEGF-A [23, 24]. Thus, we hypothesized that PERK activation promotes medulloblastoma cell migration and invasion by enhancing autocrine VEGF-A/VEGFR2 signaling.

To test this hypothesis experimentally, we first generated stably transfected medulloblastoma cell lines that allow for pharmacologically controlled activation of PERK without causing ER stress. We used the cell lines to mimic the enhancement of PERK activity to levels observed in tumor patients and determined its effects on tumor cells, thus enabling a critical evaluation of the role of PERK signaling in medulloblastoma cell migration and invasion. Our findings uncover the promoting role of PERK signaling in medulloblastoma cell migration and invasion and its underlying mechanism.

## Materials and Methods

### Cell culture

The Daoy cells were purchased from American Type Culture Collection (item number HTB-186, Manassas, VA). The UW228 cells [25] were a generous gift from Dr. John Silber (University of Washington, Seattle, WA). Both Daoy and UW228 cells were maintained in Dulbecco's Modified Eagle Medium (DMEM; Invitrogen, Carlsbad, CA) supplemented with 10% Fetal Bovine Serum (FBS, Invitrogen) and 1% penicillin/streptomycin (Invitrogen) at 37°C with 5% CO_2_. The Fv2E-PERK cDNA clone was a generous gift from Dr. David Ron (Cambridge University, Cambridge, UK). A 2.4 kb Fv2E-PERK cDNA segment was excised from the pBabe/Fv2E-PERK plasmid [26] with EcoRI and ApaI and was inserted into the multiple cloning sites region of the pcDNA3.1 plasmid (Invitrogen) at the same restriction sites. The resulting pcDNA3.1-Fv2E-PERK was further digested with EcoRI and PmeI to release the Fv2E-PERK cDNA, which was subcloned into the multiple cloning sites region of the pIREs-ZsGreen (Clontech Laboratories, Inc., Mountain View, CA) at the EcoRI and SmaI sites. Daoy cells were transfected with the mammalian expression plasmid pIREs-Fv2E-PERK-ZsGreen that contains the neomycin resistance gene using Lipofectamine 2000 transfection reagent (Invitrogen) according to the manufacturer’s instructions. The stably transfected cell lines were selected with 500 μg/ml G418 (Invitrogen).

### Western blot analysis

Near-confluent cells were treated with either AP20187 (Clontech Laboratories, Inc.), Salubrinal (EMD Millipore, Billerica, MA), GSK2606414 (EMD Millipore), or vehicle (either ethanol or DMSO) for 2 h. Cells were rinsed with ice-cold PBS and were immediately homogenized in 5 volume of Triton X-100 buffer as previously described [27, 28]. After incubating on ice for 10 min, the extracts were cleared by centrifugation at 14,000 rpm for 20 min. The protein content of each extract was determined by DC Protein Assay (Bio-Rad Laboratories, Hercules, CA). The extracts (20 μg) were separated by SDS-PAGE and were transferred to nitrocellulose. The blots were incubated with primary antibody (see below), and the signal was revealed by chemiluminescence after reacting with HRP-conjugated secondary antibody. The following primary antibodies were used: anti-FKBP-12 (polyclonal, anti-rabbit, cat# PA1–026A, 1:2000, Thermo Scientific, Rockford, IL); eIF2α (polyclonal, anti-rabbit, cat# SC-11386, 1:1000, Santa Cruz Biotechnology, Dallas, TX); phosphorylated eIF2α (p-eIF2α, anti-rabbit, cat# 9721L, 1:1000; Cell Signaling Technology, Beverly, MA), and actin (anti-rabbit, cat# A2103, 1:5000; Sigma-Aldrich, St. Louis, MO). The intensity of the recorded chemiluminescence signal was quantified using NIH ImageJ software (http://rsb. info.nih.gov/ij).

### MTT assay

Cells were plated 1 × 10^4^ per well in 96-well microtiter plates and incubated in DMEM with 10% FBS for 24 h. Then, either AP20187, SU5416 (Sigma-Aldrich), VEGF-A neutralizing antibody (NAB, R&D Systems, Minneapolis, MN), or vehicle (either ethanol, DMSO, or PBS with control mouse IgG) was added to the media and the cultures were incubated for additional 16 h. Cell viability was determined by the MTT Assay Kit (Promega, Madison, WI) according to the manufacturer's instructions.

### Real-time PCR

Near-confluent cells were treated with either AP20187, Salubrinal, or vehicle (either ethanol or DMSO) for 16 h, and then were rinsed with ice-cold PBS. RNA was isolated from cells using TRIzol reagent (Invitrogen) and was treated with DNaseI (Invitrogen) to eliminate genomic DNA. Reverse transcription was performed using the iScript cDNA Synthesis Kit (Bio-Rad Laboratories). Real-time PCR was performed with iQ Supermix (Bio-Rad Laboratories) on the LightCycler 480 System (Roche Diagnostics Corporation, Indianapolis, IN) as previously described [29–32].

### Measurement of protein synthesis with surface sensing of translation (SUnSET)

We used SUnSET to determine the rate of protein biosynthesis in cells [31, 33]. Near-confluent cells were treated with AP20187 or vehicle (ethanol). After 1 h, 1 μM puromycin (Sigma-Aldrich) was added to the cultures, and the cultures were incubated for an additional 1 h. Proteins were extracted, quantified, resolved by SDS-PAGE, and transferred to nitrocellulose. The blots were incubated with a primary antibody against puromycin (monoclonal, anti-mouse, cat# MABE343, 1:5000, EMD Millipore), followed by an HRP-conjugated secondary antibody, and following incubation with the ECL Detection Reagents (GE Healthcare Biosciences, Piscataway, NJ), the chemiluminescent signal was detected.

### Wound healing assay

Cells were cultured to confluence in 12-well plates. The confluent monolayers were scratched with a sterile 200 μl pipet tip. The cells were raised with PBS, and then incubated in DMEM with 0.5% FBS and various treatments. Images were taken by phase contrast microscopy using the 10 × objective at the beginning (0 h) and after 16 h of incubation. The wound areas were quantified at 0 h and after 16 h of incubation using NIH Image J program (http://imagej.nih.gov/ij/). The percentage of filled wound area was calculated as the filled wound area at 16 h (the wound area at 0 h—the wound area at 16 h) divided by the wound area at 0 h.

### Matrigel transwell assay

The cell culture chambers (8 μm pore membranes, BD Biosciences, Bedford, MA) inserted in a 24-well plate were coated with matrigel (300 μg/ml, BD Biosciences). 0.1–1 × 10^5^ cells were plated in the top well of a matrigel-coated chamber. Both the top well and the lower well of the chamber were filled with DMEM with 0.5% FBS and various treatments. After 16 h of incubation, non-invading cells were removed with a cotton swab and the invading cells that had crossed the matrigel to the underside of the membrane were stained with crystal violet and photographed. The stained cells were dissolved in 10% acetic acid and the absorbance was measured at 561 nm.

### Enzyme-linked immunosorbent assay (ELISA)

Cells were plated 1 × 10^5^ per well in 12-well plates. After 24 h, the cells were raised with PBS, and then incubated in DMEM with 0.5% FBS and various treatments for 16 h. We quantified VEGF-A in the culture supernatants of the wells using human VEGF-A ELISA kit (Thermo Scientific) according to the manufacturer’s instructions.

### Statistics

Data are expressed as mean ± standard deviation (SD). Multiple comparisons were statistically evaluated by a 1-way ANOVA test using Sigmaplot 12 software (Systat Software, Inc., Chicago, IL). *P* values less than 0.05 were considered significant.

## Results

### Moderate PERK activation promotes medulloblastoma cell migration and invasion

**Fig 1:**
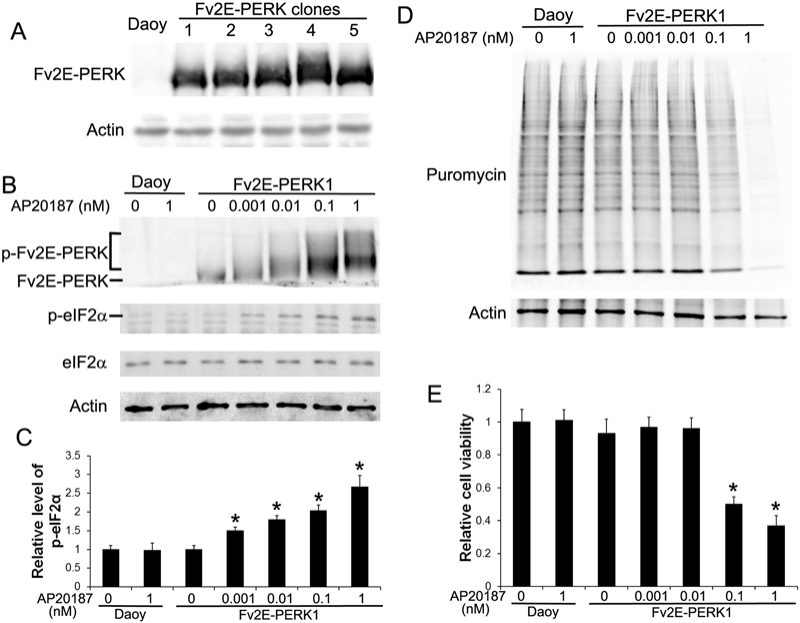
Characterization of stably transfected Daoy cell lines that allow for pharmacological controlled activation of PERK. (A) Daoy cells were transfected with the plasmid pIREs-Fv2E-PERK-ZsGreen. We obtained several stably transfected cell lines that were resistant to G418 and expressed various levels of Fv2E-PERK. (B) Western blot analysis showed that AP20187 treatment activated Fv2E-PERK and led to phosphorylation of eIF2α. The positions of activated Fv2E-PERK (p-Fv2E-PERK) and inactive Fv2E-PERK proteins were indicated. (C) Densitometry analysis of western blot results showed that AP20187 treatment increased the level of p-eIF2a in Fv2E-PERK1 cells in a dose-dependent manner. The relative protein levels are relative to actin. (D) SUnSET measurement of protein biosynthesis revealed dramatic reduction of protein biosynthesis in Fv2E-PERK1 cells treated with the high dose of AP20187 (0.1–1 nM). Nevertheless, treatment with the low dose of AP20187 (0.001–0.01 nM) only slightly reduced protein biosynthesis in the cells. (E) MTT analysis showed that treatment with the high dose of AP20187 (0. 1–1 nM) significantly inhibited Fv2E-PERK1 cell growth. Nevertheless, treatment with the low dose of AP20187 (0.001–0.01 nM) had no effect on the cell growth. The experiments were repeated at least three times. Error bars represent SD, *P < 0.05.

**Fig 2:**
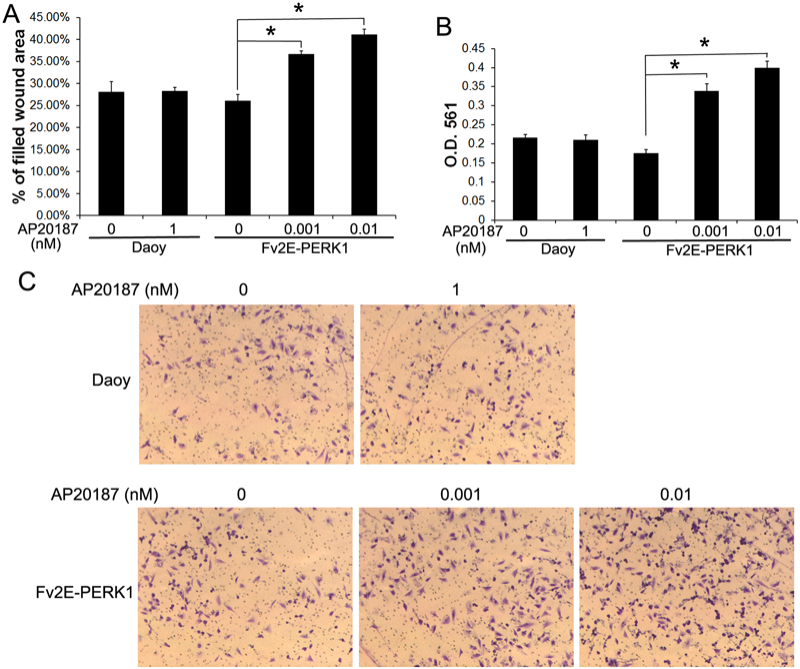
Moderate PERK activation promoted Daoy cell migration and invasion. (A) Wound healing assay showed that AP20187 (0.001–0.01 nM) treatment significantly increased the percentage of filled wound area of Fv2E-PERK1 cells. (B, C) Results of matrigel transwell assay. Daoy or Fv2E-PERK1 cells were seeded in the top well of a matrigel-coated chamber. After 16 h, the invaded cells at the lower surface of the chamber were stained with crystal violet and photographed (C). The stained cells were dissolved in 10% acetic acid and the absorbance was measured at 561 nm (B). AP20187 (0.001–0.01 nM) treatment significantly increased the invaded Fv2E-PERK1 cell numbers at the lower surface of the chamber. The experiments were repeated at least three times. Error bars represent SD, *P < 0.05.

The three functional domains of PERK are: a stress-sensing ER luminal domain, an ER transmembrane domain, and a cytosolic eIF2α kinase domain [34]. During ER stress, the stress-sensing ER lumenal domain senses the stress signal and initiates PERK oligomerization and autophosphorylation, resulting in activation of PERK signaling. Fv2E-PERK, an artificial PERK derivative, is generated by fusing the eIF2α kinase effector domain of PERK to a polypeptide containing tandem modified FK506 binding domains (Fv2E). It has been demonstrated that Fv2E-PERK can be induced to oligomerize and activate by an otherwise inert ligand, AP20187, in the absence of any additional stress [26]. We transfected human medulloblastoma cell line Daoy with plasmid pIREs-Fv2E-PERK-ZsGreen and obtained several stably transfected cell lines that were resistant to G418 and expressed various levels of Fv2E-PERK (Fig. 1A). The effects of overexpression of Fv2E-PERK alone on Daoy cell growth, migration, and invasion were determined. We found that stably transfected Daoy cell line # 1 (Fv2E-PERK1) expressed a high level of Fv2E-PERK and that overexpression of Fv2E-PERK alone did not significantly affect the cell growth, migration, and invasion as compared to control Daoy cells (Figs. 1, 2). Thus, Fv2E-PERK1 cell line was selected for all subsequent studies.

**Fig 3:**
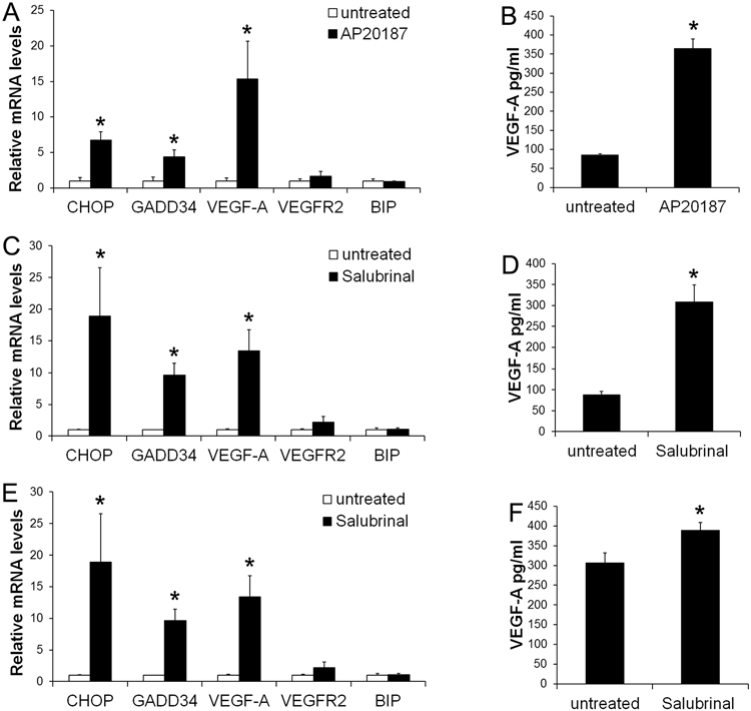
PERK activation stimulated medulloblastoma cells to produce VEGF-A. (A) Real-time PCR analysis showed that 0.01 nM AP20187 treatment significantly increased the expression of CHOP, GADD34, and VEGF-A in Fv2E-PERK1 cells, but did not affect the expression of VEGFR2 and BIP. (B) ELISA analysis showed that 0.01 nM AP20187 treatment significantly increased the production of VEGF-A in Fv2E-PERK1 cells. (C) Real-time PCR analysis showed that 10 μM Salubrinal treatment significantly increased the expression of CHOP, GADD34, and VEGF-A in Daoy cells, but did not affect the expression of VEGFR2 and BIP. (D) ELISA analysis showed that 10 μM Salubrinal treatment significantly increased the production of VEGF-A in Daoy cells. (E) Real-time PCR analysis showed that 10 μM Salubrinal treatment significantly increased the expression of CHOP, GADD34, and VEGF-A in UW228 cells, but did not affect the expression of VEGFR2 and BIP. (F) ELISA analysis showed that 10 μM Salubrinal treatment significantly increased the production of VEGF-A in UW228 cells. The experiments were repeated at least three times. Error bars represent SD, *P < 0.05.

Importantly, we found that treatment with Fv2E ligand AP20187 had no effect on control Daoy cells (Figs. 1, 2), but triggered the transgenic product Fv2E-PERK activation in Fv2E-PERK1 cells in a dose-dependent manner. Western blot analysis using the anti-FKBP-12 antibody, which recognizes the modified FK506 binding domain and has been successfully used to detect both the unphosphorylated and phosphorylated forms of Fv2E-PERK [8, 29], showed that treatment with AP20187 (0.001–1 nM) triggered phosphorylation of Fv2E-PERK in Fv2E-PERK1 cells in a dose-dependent manner (Fig. 1B). PERK activation inhibits global protein translation by phosphorylating eIF2α. Western blot analysis for p-eIF2α also showed that AP20187 (0.001–1 nM) treatment elevated the level of p-eIF2α in the cells in a dose-dependent manner (Fig. 1B, C). Moreover, we assessed the activity of the PERK-eIF2α pathway in Fv2E-PERK1 cells by measuring the rate of protein biosynthesis using SUnSET, a puromycin-based technology. Interestingly, we found that treatment with the high dose of AP20187 (0.1–1 nM) dramatically reduced protein biosynthesis in Fv2E-PERK1 cells; however, treatment with the low dose of AP20187 (0.001–0.01 nM) only slightly reduced protein biosynthesis (Fig. 1D). As expected, real-time PCR analysis showed that AP20187 treatment increased the expression of PERK responsible genes CAATT enhancer binding protein homologous protein (CHOP) and GADD34, but did not significantly affect the expression of binding immunoglobulin protein (BIP) (Fig. 3A). Previous reports have shown that induction of CHOP and GADD34 was completely dependent on the activity of PERK during ER stress and the effect of PERK signaling on BIP induction was minimal [29–31, 35]. Therefore, these data suggest that AP20187 treatment exclusively enhances the activity of PERK in Fv2E-PERK1 cells without affecting the other branches of the UPR. Additionally, MTT assay showed that the growth of Fv2E-PERK1 cells was significantly inhibited by the high dose of AP20187 (0.1–1 nM) treatment, but not by the low dose of AP20187 (0.001–0.01 nM) treatment (Fig. 1E). Taken together, these results suggest that the high dose of AP20187 treatment strongly activates PERK signaling in Fv2E-PERK1 cells and inhibits cell growth. Nevertheless, the low dose of AP20187 treatment moderately activates PERK signaling in Fv2E-PERK1 cells, but does not affect cell growth. It is generally believed that ER stress and the UPR activation under physiological and pathological conditions are moderate [31, 36]. Thus, to mimic the moderate PERK activation under pathological conditions, Fv2E-PERK1 cells were treated with the low dose of AP20187 (0.01 nM) for all subsequent studies.

**Fig 4:**
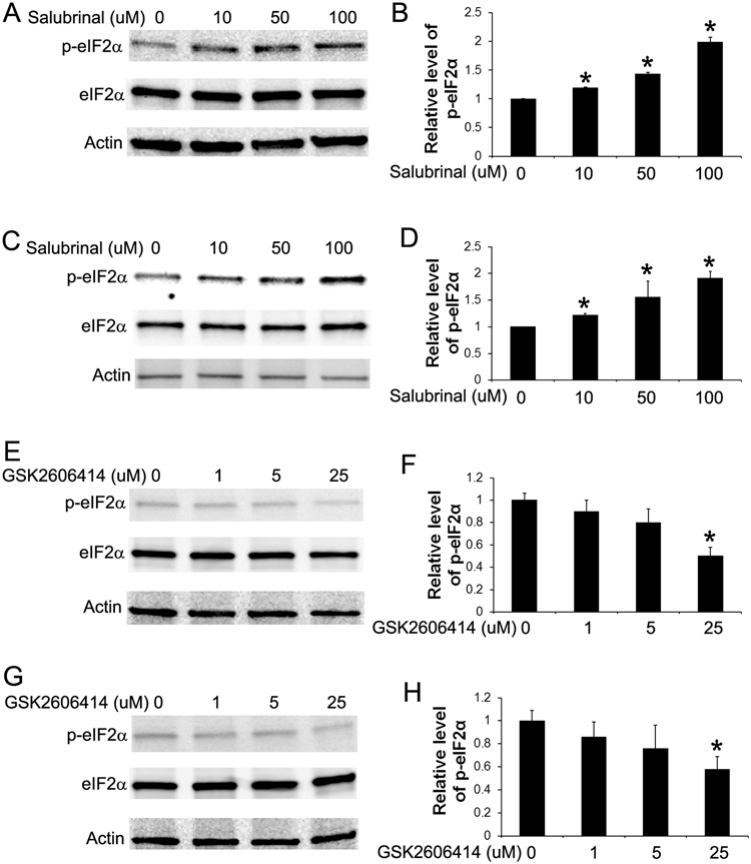
Treatment with either Salubrinal or GSK2606414 altered the levels of p-eIF2α in medulloblastoma cells. (A, B) Western blot analysis showed that treatment with Salubrinal (10–100 μM) moderately but significantly elevated the level of p-eIF2α in Daoy cells. (C, D) Western blot analysis showed that treatment with Salubrinal (10–100 μM) moderately but significantly elevated the level of p-eIF2α in UW228 cells. (E, F) Western blot analysis showed that treatment with GSK2606414 (25 μM) noticeably decreased the level of p-eIF2α in Daoy cells. (G, H) Western blot analysis showed that treatment with GSK2606414 (25 μM) noticeably decreased the level of p-eIF2α in UW228 cells. The relative protein levels are relative to actin. The experiments were repeated at least three times. Error bars represent SD, *P < 0.05.

**Fig 5:**
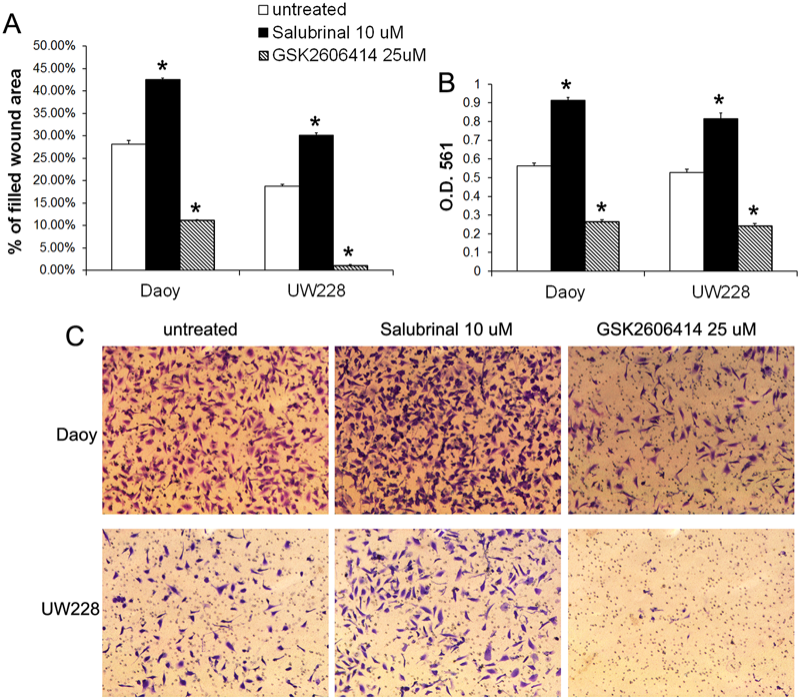
Moderate PERK activation enhanced medulloblastoma cell migration and invasion. (A) Wound healing assay showed that Salubrinal (10 μM) treatment significantly increased the percentage of filled wound area of Daoy and UW228 cells, and that GSK2606414 (25 μM) treatment significantly decreased the percentage of filled wound area of Daoy and UW228 cells. (B, C) Matrigel transwell assay showed that Salubrinal (10 μM) treatment significantly increased the numbers of the invaded Daoy and UW228 cells at the lower surface of the chamber, and that GSK2606414 (25 μM) treatment significantly decreased the numbers of the invaded Daoy and UW228 cells at the lower surface of the chamber. The experiments were repeated at least three times. Error bars represent SD, *P < 0.05.

Next, we determined the effects of moderate PERK activation on medulloblastoma cell migration and invasion. Wound healing assay showed that treatment with the low dose of AP20187 (0.001–0.01 nM) significantly increased the migration capacity of Fv2E-PERK1 cells into the wounds (Fig. 2A). Matrigel transwell assay showed that the low dose of AP20187 (0.001–0.01 nM) treatment significantly enhanced the capacity of Fv2E-PERK1 cells to invade through the matrigel (Fig. 2B, C). Finally, we verified the effects of moderate PERK activation on medulloblastoma cell migration and invasion using a pharmacologic approach. Medulloblastoma cell lines, Daoy and UW228, were treated with either Salubrinal, a small chemical compound that inhibits the activity of the phosphatase complex that dephosphorylates eIF2α [37, 38], or GSK2606414, a highly specific small molecule inhibitor of PERK kinase [39, 40]. Western blot analysis showed that treatment with Salubrinal (10–100 μM) moderately but significantly increased the level of p-eIF2α in both Daoy and UW228 cells (Fig. 4A, B, C, D). Real-time PCR analysis showed that Salubrinal treatment increased the expression of CHOP and GADD34 in both Daoy and UW228 cells, but did not significantly affect the expression of BIP (Fig. 3C, E). In contrast, treatment with GSK2606414 (25 μM) noticeably decreased the level of p-eIF2α in both Daoy and UW228 cells (Fig. 4E, F, G, H). Wound healing assay showed that treatment with the low dose of Salubrinal (10 μM) significantly facilitated Daoy and UW228 cell migration, and that treatment with GSK2606414 (25 μM) significantly suppressed Daoy and UW228 cell migration (Fig. 5A). Matrigel transwell assay showed that 10 uM Salubrinal treatment significantly promoted the invasion of Daoy and UW228 cells, and that 25 μM GSK2606414 treatment significantly inhibited the invasion of Daoy and UW228 cells (Fig. 5B, C). Collectively, these data suggest that moderate activation of the PERK-eIF2α pathway promotes medulloblastoma cell migration and invasion.

### Tumor cell-derived VEGF-A promotes medulloblastoma cell migration and invasion

**Fig 6:**
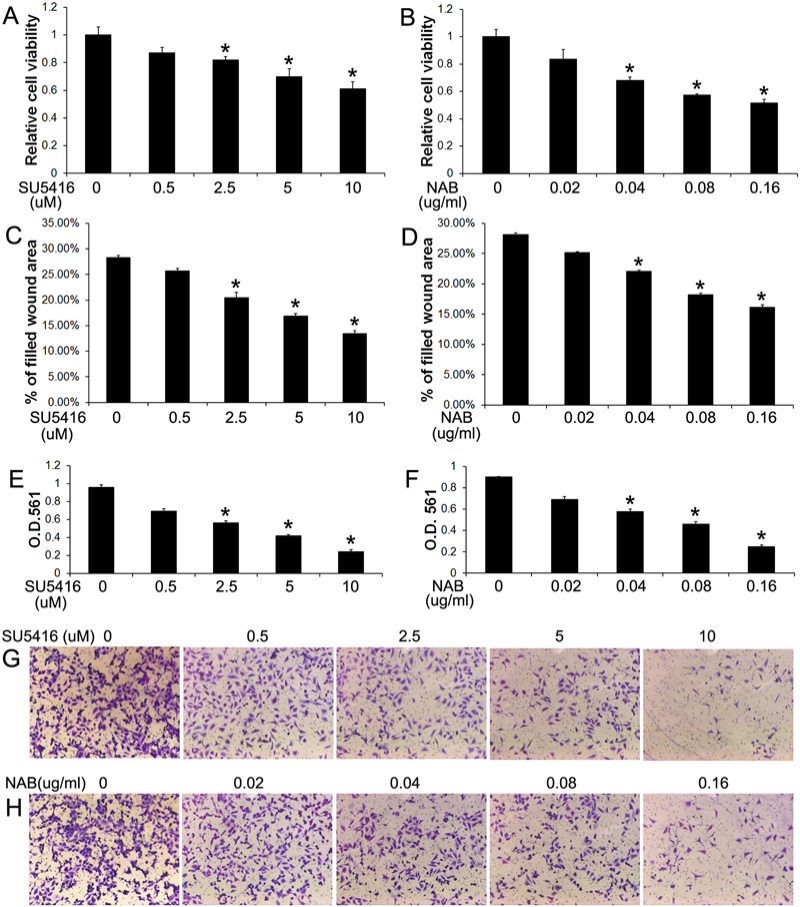
Blockage of VEGF-A impaired medulloblastoma cell migration and invasion. (A) MTT assay showed that SU5416 treatment significantly suppressed Daoy cell growth. (B) MTT assay showed that NAB treatment significantly suppressed Daoy cell growth. (C) Wound healing assay showed that SU5416 treatment significantly reduced the percentage of filled wound area of Daoy cells. (D) Wound-healing assay showed that NAB treatment significantly reduced the percentage of filled wound area of Daoy cells. (E, G) Matrigel transwell assay showed that SU5416 treatment significantly reduced the numbers of the invaded Daoy cells at the lower surface of the chamber. (F, H) Matrigel transwell assay showed that NAB treatment significantly reduced the numbers of the invaded Daoy cells at the lower surface of the chamber. The experiments were repeated at least three times. Error bars represent SD, *P < 0.05.

**Fig 7:**
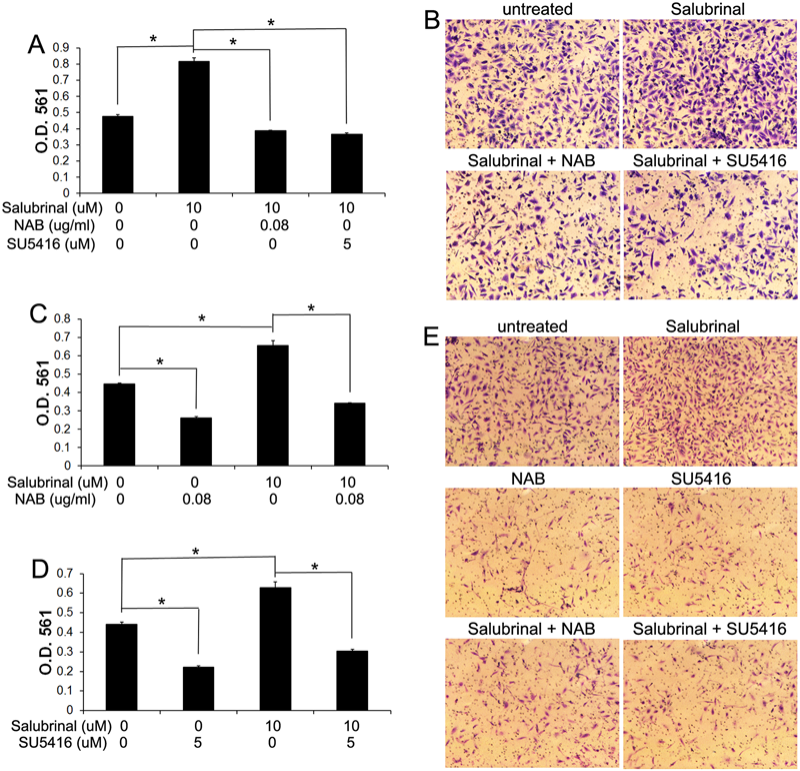
PERK activation promoted medulloblastoma cell invasion through activation of VEGF-A/VEGFR2 signaling. (A, B) Matrigel transwell assay showed that 10 μM Salubrinal treatment significantly increased the numbers of the invaded Daoy cells at the lower surface of the chamber. Interestingly, treatment with either 0.08 μg/ml NAB or 5 μM SU5416 diminished the increased cell number at the lower surface of the chamber induced by Salubrinal treatment. (C, E) Matrigel transwell assay showed that 10 μM Salubrinal treatment significantly increased the numbers of the invaded UW228 cells at the lower surface of the chamber; however, treatment with 0.08 μg/ml NAB significantly reduced the numbers of the invaded UW228 cells at the lower surface of the chamber. Interestingly, treatment with 0.08 μg/ml NAB also diminished the increased cell number at the lower surface of the chamber induced by Salubrinal treatment. (D, E) Matrigel transwell assay showed that treatment with 5 μM SU5416 significantly reduced the numbers of the invaded UW228 cells at the lower surface of the chamber. Importantly, 5 μM SU5416 treatment also diminished the increased cell number at the lower surface of the chamber induced by Salubrinal treatment. The experiments were repeated at least three times. Error bars represent SD, *P < 0.05.

A previous report has shown that medulloblastoma cells express VEGF-A and VEGFR2 and that treatment with a VEGFR2 inhibitor, SU5416, suppresses the cell growth [22]. Consistent with this report, we found that medulloblastoma cell lines, Daoy and UW228, expressed VEGF-A and VEGFR2 (Fig. 3), and that treatment with SU5416 (2.5–10 μM) significantly suppressed the growth of Daoy cells (Fig. 6A). Interestingly, wound healing assay showed that treatment with SU5416 (2.5–10 μM) resulted in a significant decrease in the migration capacity of Daoy cells (Fig. 6C), as assessed by the percentage of filled wound area after 16 h of cell migration. Matrigel transwell assay showed that treatment with SU5416 (2.5–10 μM) led to a significant reduction in the invasion capacity of Daoy cells (Fig. 6E, G) and UW228 cells (Fig. 7D, E), as assessed by the number of cells that invaded through the matrigel. Thus, using the pharmacologic approach, our data suggest that VEGFR2 signaling is involved in medulloblastoma migration and invasion.

The activity of VEGFR2 can be regulated by a number of ligands, including VEGF-A [41]. To determine the autocrine role of VEGF-A in medulloblastoma migration and invasion, we further exploited a VEGF-A neutralizing antibody (NAB). We found that treatment with the NAB (0.04–0.16 μg/ml) also significantly inhibited the growth of Daoy cells (Fig. 6B). Wound healing assay showed that treatment with the NAB (0.04–0.16 μg/ml) significantly reduced the migration capacity of Daoy cells (Fig. 6D). Furthermore, matrigel transwell assay showed that treatment with the NAB (0.04–0.16 μg/ml) significantly decreased the invasion capacity of Daoy cells (Fig. 6F, H) and UW228 cells (Fig. 7C, E). Thus, using the VEGFR2 inhibitor SU5416 and the NAB to block VEGF-A/VEGFR2 signaling, our data suggest that VEGF-A acts directly on medulloblastoma cells in an autocrine manner to promote cell migration and invasion.

### VEGF-A is required for the promoting effects of moderate PERK activation on medulloblastoma cell migration and invasion

Several studies have shown that PERK activation increased VEGF-A expression in medulloblastoma cells [18, 23, 24]. We determined the involvement of VEGF-A in the promoting effects of PERK activation on medulloblastoma cell migration and invasion. As expected, real-time PCR analysis showed that 0.01 nM AP20187 treatment significantly elevated the mRNA levels of CHOP, GADD34, and VEGF-A, in Fv2E-PERK1 cells, but did not significantly affect the expression of VEGFR2 (Fig. 3A). ELISA measurement of VEGF-A production in culture supernatants showed that 0.01 nM AP20187 treatment stimulated Fv2E-PERK1 cells to secrete VEGF-A (Fig. 3B). Similarly, we found that 10 μM Salubrinal treatment resulted in the significant elevated mRNA levels of CHOP, GADD34, and VEGF-A in Daoy and UW228 cells, but had no effects on the VEGFR2 mRNA level (Fig. 3C, E). Moreover, ELISA measurement of VEGF-A production in culture supernatants revealed that 10 μM Salubrinal treatment significantly enhanced the secretion of VEGF-A in both Daoy and UW228 cells (Fig. 3D, F).

**Fig 8:**
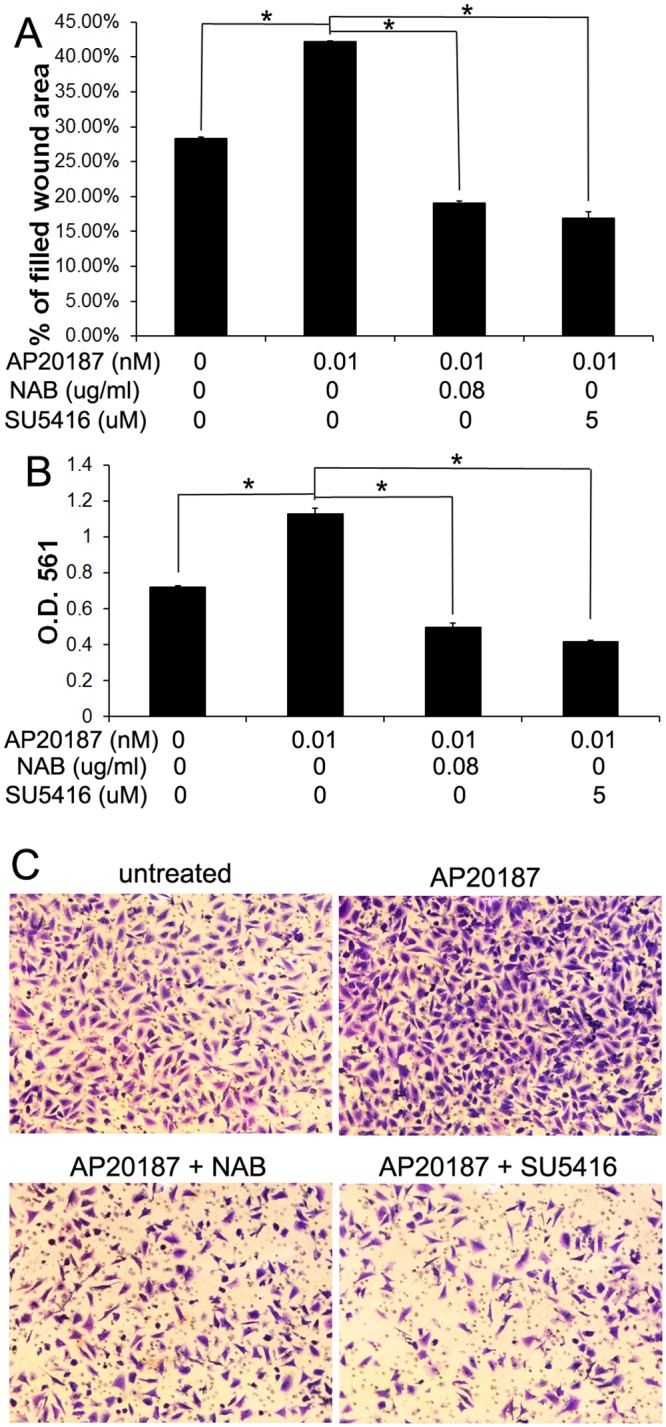
VEGF-A contributed to the promoting effects of PERK activation on Daoy cell migration and invasion. (A) Wound-healing assay showed that 0.01 nM AP20187 treatment significantly increased the percentage of filled wound area of Fv2E-PERK1 cells. Importantly, the increased percentage of filled wound area induced by AP20187 treatment was diminished by treatment with either 0.08 μg/ml NAB or 5 μM SU5416. (B, C) Matrigel transwell assay showed that 0.01 nM AP20187 treatment significantly increased the numbers of the invaded Fv2E-PERK1 cells at the lower surface of the chamber. Interestingly, treatment with either 0.08 μg/ml NAB or 5 μM SU5416 diminished the increased cell number at the lower surface of the chamber induced by AP20187 treatment. The experiments were repeated at least three times. Error bars represent SD, *P < 0.05.

Wound healing assay showed that 0.01 nM AP20187 treatment significantly promoted Fv2E-PERK1 cell migration; however, 0.08 μg/ml NAB treatment diminished the ability of AP20187 treatment to promote cell migration (Fig. 8A). Matrigel transwell assay showed that 0.01 nM AP20187 treatment significantly increased the invaded Fv2E-PERK1 cell numbers at the lower surface of the chamber, and that 0.08 μg/ml NAB abrogated the increased number of invaded cells induced by AP20187 treatment (Fig. 8B, C). Moreover, matrigel transwell assay showed that 10 μM Salubrinal treatment significantly increased the invaded cell numbers of Daoy and UW228 at the lower surface of the chamber, and that 0.08 μg/ml NAB treatment impaired the ability of Salubrinal treatment to increase the number of invaded cells (Fig. 7A, B, C, E). Collectively, these data suggest that moderate PERK activation promotes medulloblastoma cell migration and invasion through induction of VEGF-A.

Furthermore, using the VEGFR2 inhibitor SU5416, our data suggested that VEGFR2 was required for the promoting effects of PERK activation on medulloblastoma cell migration and invasion. We found that the ability of 0.01 nM AP20187 treatment to promote Fv2E-PERK1 cell migration and invasion was diminished by treatment with 5 μM SU5416, as shown by wound-healing assay and matrigel transwell assay (Fig. 8), respectively. Additionally, matrigel transwell assay showed that 5 μM SU5416 treatment diminished the ability of 10 μM Salubrinal treatment to promote Daoy and UW228 cell invasion (Fig. 7). Thus, using the VEGFR2 inhibitor SU5416 and the NAB to block VEGF-A/VEGFR2 signaling, our results suggest that moderate PERK activation promotes medulloblastoma cell migration and invasion through activation of VEGF-A/VEGFR2 signaling.

## Discussion

It is well documented that the PERK branch of the UPR is activated in solid tumors [5, 6, 9]. Recent reports suggest that activation of PERK signaling contributes to the promoting effects of hypoxia on breast cancer cell migration and invasion [12, 42]. In this study, our findings provided evidence that moderate activation of the PERK-eIF2α pathway enhanced medulloblastoma cell migration and invasion through activation of VEGF-A/VEGFR2 signaling. We found that moderately enhanced PERK activation, via a genetic approach or a pharmacologic approach, facilitated medulloblastoma cell migration and invasion and increased the production of VEGF-A. Moreover, using the VEGFR2 inhibitor SU5416 and the NAB to block VEGF-A/VEGFR2 signaling, our results suggested that both VEGF-A and its receptor VEGFR2 were required for the promoting effects of PERK activation on medulloblastoma cell migration and invasion.

It is believed that PERK activation has biphasic effects on cell viability, dependent on the degree of activation [2, 43, 44]. Strong PERK activation in response to severe ER stress is detrimental to cells through inhibition of global protein translation and/or induction of CHOP, a pro-apoptotic transcription factor [8, 13, 31]. In contrast, moderate PERK activation adapts cells to mild ER stress under physiological and pathological conditions [8, 29, 30]. Herein, we used a genetic tool to generate stably transfected medulloblastoma cell lines that allow for dose-dependent activation of PERK by the small chemical compound AP20187, in the absence of ER stress. We showed that strong PERK activation induced by the high dose of AP20187 treatment severely inhibited protein translation in the medulloblastoma cells and suppressed cell growth. Not surprisingly, we also found that strong PERK activation suppressed the medulloblastoma cell migration and invasion (unpublished data). In contrast, moderately enhanced PERK activation induced by the low dose of AP20187 treatment slightly reduced protein translation in the medulloblastoma cells and had no significant effect on cell growth. Interestingly, moderately enhanced PERK activation promoted the medulloblastoma cell migration and invasion. Moreover, we found that moderately enhanced PERK activation induced by Salubrinal treatment enhanced medulloblastoma cell migration and invasion and that impaired PERK activation induced by GSK2606414 treatment suppressed medulloblastoma cell migration and invasion. Taken together, our data suggest that the effects of PERK activation on tumor cell migration and invasion are also dependent on the degree of activation, namely that moderate PERK activation is promoting, whereas strong PERK activation is suppressing.

VEGF-A is thought to play a critical role in tumor progression through enhancing angiogenesis [19]. Angiogenesis not only enhances tumor growth by supplying more nutrients and oxygen to the tumors, but also increases the opportunity for tumor cells to enter the circulation and disseminate. Moreover, there is evidence that VEGF-A can act directly on some tumor cell types in an autocrine manner to promote tumor growth and invasion [20, 21]. A number of studies reveal the elevated levels of VEGF-A and VEGFR2 in medulloblastoma of human patients [45, 46]. Recent studies also show that medulloblastoma cells express both VEGF-A and VEGFR2 [22, 47]. Nevertheless, the role of VEGF-A that plays in medulloblastoma remains elusive. A study reported that VEGF-A promotes medulloblastoma cell growth via VEGFR2 signaling [22]. Other studies, however, showed that VEGF-A has no effect on medulloblastoma cell migration and invasion [47, 48]. Using two medulloblastoma cell lines, Daoy and UW228, we demonstrated that these cell lines expressed both VEGF-A and VEGFR2. To determine the effects of VEGF-A/VEGFR2 signaling on medulloblastoma, we selected the well-documented VEGFR2 inhibitor SU5416 [22, 49] and the well-documented NAB [50, 51] to manipulate the activity of this pathway in medulloblastoma cells. As expected, we showed that treatment with either the NAB or the VEGFR2 inhibitor suppressed medulloblastoma cell growth when the cells were incubated in DMEM with 10% FBS. We also found that treatment with either the NAB or the VEGFR2 inhibitor had no significant effects on medulloblastoma cell viability under serum starvation conditions (unpublished data). Importantly, we showed that treatment with either the NAB or the VEGFR2 inhibitor suppressed medulloblastoma cell migration and invasion under serum starvation conditions. Therefore, these results suggest that VEGF-A acts directly on medulloblastoma cells in an autocrine manner to promote tumor migration and invasion. Unfortunately, we could not provide direct evidence for the autocrine role of VEGF-A/VEGFR2 signaling in medulloblastoma cells using a genetic approach, namely the RNA interference approach. Despite considerable efforts, we were not capable of establishing medulloblastoma cell lines that were stably transfected with VEGFR2 short hairpin RNA (shRNA). The possibilities that account for the contradictory results regarding the effects of VEGF-A on medulloblastoma cells could include the following: 1. different cell lines were used, Akino et al. [48] showed that VEGF-A did not affect D283, D425, D458 and D556 cell migration and invasion because these cell lines do not express VEGFR2; 2. different experimental approaches were used, Davare et al. [47] showed that VEGF-A did not influence Daoy cell invasion because VEGF-A was added to the culture media in the lower chamber as chemoattractant.

After tumor cells accumulate mutations that overcome cell cycle and apoptotic checkpoints, they must acquire the abilities to adapt to the tumor microenvironment, to invade, and to promote angiogenesis, which is thought to be the critical step in tumor progression [52, 53]. Nevertheless, little is known about how medulloblastoma cells acquire these abilities. It is well documented that activation of the PERK branch of the UPR adapts tumor cells to the tumor microenvironment [10, 54, 55]. We showed here that moderately enhanced PERK activation promoted medulloblastoma cell migration and invasion, and increased the expression of VEGF-A, a master regulator of angiogenesis. Importantly, using the VEGFR2 inhibitor SU5416 and the NAB to block VEGF-A/VEGFR2 signaling, our results suggested that VEGF-A was required for the promoting effects of PERK activation on medulloblastoma cell migration and invasion. Taken together, these data imply that moderate PERK activation contributes to the abilities of medulloblastoma cells to adapt to the tumor microenvironment, to invade, and to promote angiogenesis. Furthermore, the results presented in this study indicate the role of VEGF-A in the promoting effects of moderate PERK activation on medulloblastoma invasion and angiogenesis.
